# Universal health coverage of five essential health services in mothers before and after the Haiti 2010 earthquake: a retrospective cohort study using difference-in-difference

**DOI:** 10.1186/s12913-022-08896-1

**Published:** 2022-12-10

**Authors:** Naoki Hirose, Sanmei Chen, Koichiro Shiba, Crystal L. Patil, Md Moshiur Rahman, Yoko Shimpuku

**Affiliations:** 1 Global Health Nursing, Graduate School of Biomedical and Health Sciences, Hiroshima University, 1-2-3, Kasumi, Minamiku, Hiroshima, 734-8553 Japan; 2 School of Public Health, Boston University, Boston, MA USA; 3 University of Illinois Chicago, Chicago, IL USA

**Keywords:** Universal Health Coverage, Earthquake, Haiti, Reproductive health, Difference-in-difference

## Abstract

**Background:**

In January 2010, Haiti was hit by a 7.0-magnitude earthquake. The impact of the earthquake on Universal Health Coverage in mothers remains unclear. This study explores the association between the 2010 Haiti earthquake and access to the five quality essential health services among women who gave birth in the two years before and after the earthquake.

**Methods:**

From the Sixth Demographic and Health Survey in Haiti, we extracted data for women aged 15–49 who had reported a live birth in the two years before and after the 2010 Haiti earthquake. We used difference-in-difference analyses for antenatal care, delivery care, and vaccination, and multivariate logistic regression analyses for family planning and malaria prevention, to assess the impact of the acute damage (household-level damage, such as housing damage and/or loss of a family member, or region-level damage, such as living in a region where 50% or more of the houses were damaged) of the earthquake on these mothers’ access to quality essential health services.

**Results:**

Mothers who had not suffered acute earthquake damage were more likely to live in rural areas and had less education and household wealth. The difference-in-difference and multivariate logistic regression analyses did not show strong evidence of any significant association between acute earthquake damage and access to quality health services. However, after the earthquake, access to quality health services deteriorated for both mothers with and without acute earthquake damage (-5.6% and -6.2% for antenatal care, -6.5% and 0% for delivery care, and -9.5% and -13.1% for vaccination, respectively).

**Conclusions:**

The earthquake adversely affected mothers’ access to quality essential health services regardless of their exposure to acute earthquake damage. Mothers in rural areas who avoided such damage might also have experienced long-term negative effects from the earthquake, which was likely exacerbated by other structural factors such as lower education and economic status.

## Introduction

### Background

Universal health coverage (UHC), endorsed by the World Health Organization (WHO), means that all people receive the quality essential health services that they need without suffering from financial hardship [1]. Since the 58^th^ World Health Assembly resolution in 2005, the importance of UHC has been widely recognized, and in 2015, UHC was counted as one of the United Nations Sustainable Development Goals [1].

Achieving or sustaining UHC even in the context of the emergency (e.g., a major natural disaster) is imperative to minimizing the suffering related to disrupted access to essential health services, especially for vulnerable populations, such as mothers and infants [2]. For example, a devastating earthquake affects the health system both in the short term and long term. In the short term, it kills people, destroys homes and health facilities, and devastates water and communications supplies [3]. In the long term, it might cause the spread of infectious diseases due to a breakdown of sanitation, high cost to rebuild the health care system, and lack of health care professionals and health supplies.

In January 2010, Haiti was hit by a 7.0-magnitude earthquake, causing an estimated 316,000 deaths and 300,000 injuries, displacing 1.3 million people, and destroying 97,294 houses [4]. In Port-au-Price, the capital city of Haiti, the earthquake destroyed and/or damaged 8 out of 11 major public hospitals [5]. This destruction of health facilities led to delays in delivering health care services to those who suffered. Previous studies indicated that some of the essential health services for mothers and babies were disrupted by this devastating earthquake [6–8]. For example, in addition to increase in the infant mortality and child mortality rates, mothers’ exposure to this earthquake was associated with multiple adverse health events, including increased risk for intrauterine growth restriction, reduced use of injectables for family planning, and increased unplanned pregnancies. However, previous studies listed above focused on pregnancy and birth outcomes and accessibility to essential health services, but the quality of these health services, a key indicator of UHC, remained unexplored, even though quality is a critical factor in the desired health outcomes [6–8]. Also, these studies focused on limited essential health services in UHC such as family planning, malaria prevention, or vaccination, even though the earthquake might have impacted other essential health services, such as antenatal care and delivery care.

To achieve and sustain UHC even under an emergency like an earthquake, it is critical to identify the weaknesses in the system that cause limited access to quality services for the wide array of essential health services for mothers. In this study, we explored associations between the 2010 Haiti earthquake and the access to quality health services in family planning, antenatal care, delivery care, vaccination, and malaria prevention. We hypothesize that the mothers exposed to acute damage from the earthquake, such as household-level housing damage and/or loss of a family member or region-level collapse of houses, had more deteriorated access to quality health services compared to mothers who did not.

## Methods

### Data source

We used the Sixth Demographic and Health Survey (DHS) in Haiti, which comprised retrospective data collected after the earthquake for 14,287 women aged 15–49. The DHS is a nationally representative household survey conducted in more than 85 countries worldwide since 1984. DHS has high response rates (typically more than 90%) [9], nationwide coverage, highly quality training for interviewer, and standardized data collection across regions and countries over time. In DHS data collection process, trained interview teams visit randomly selected households during the interview period (between January and June 2012 for the Sixth Haiti DHS) and conduct interviews with eligible household members.

We included women who reported a live birth in the two years before and after the 2010 Haiti earthquake (i.e., between January 2008 and June 2012). We included only the latest birth if a woman had multiple births because the DHS collected detailed data on pregnancy or delivery of the latest birth. The women who reported a live birth more than two years before the earthquake were excluded to minimize potential recall bias. We applied additional inclusion and exclusion criteria to make the sub-cohort for each outcome because the mothers’ need for health services were different depending on the type of services (Table 1).

**Table 1:** Inclusion and exclusion criteria and definition of each outcome of the health care services

	Cohort criteria	Outcome definition
Family planning	Inclusion criteria (all of the following) 1. Currently married 2. 15–49 years old 3. Currently not pregnant 4. Does not want another child now or ever Exclusion criteria (any of the following) 1. Never menstruated or menstruated before last birth 2. In menopause or had hysterectomy 3. Declared infecund 4. Never had sex 5. Using modern contraceptive method not related with side effects (further questions about side effects were not collected for those methods)	Outcome domains (threshold: 3 or more) 1. Currently using modern contraceptive methods (IUD, injectables, implants, or pill) 2. Informed about the side effects of these methods 3. Informed about how to deal with the side effects 4. Informed about other family planning methods
Antenatal care	Inclusion criteria (all of the following) 1. 15–49 years old 2. Have at least one birth in 5 years 3. Birth after 2008	Outcome domains (threshold: 5 or more) 1. First ANC visit in up to 12 weeks 2. More than 4 ANC visits 3. Blood pressure taken during pregnancy 4. Urine sample taken during pregnancy 5. Blood sample taken during pregnancy 6. Had taken drugs for intestinal worms during pregnancy 7. Were informed about signs suggesting problem in pregnancy 8. Were given iron tablet during pregnancy
Delivery care	Inclusion criteria (all of the following) 1. 15–49 years old 2. Have at least one birth in 5 years 3. Birth after 2008 Exclusion criteria (any of the following) 1. Child death within 2 months 2. Birth after May 2012 (at least 2-month follow-up period)	Outcome component (threshold: 4 or more) 1. Received health check for mother after delivery 2. Received health check for baby within 1 h after delivery 3. Stayed at health facility after birth for at least 24 h 4. Receiving the health check for baby within 2 months after delivery 5. Receiving a vitamin A dose in 2 months after delivery 6. Ever breastfed 7. Baby was placed at mother's breast within 60 min after delivery
Vaccination	Inclusion criteria (all of the following) 1. 15–49 years old 2. Have at least one birth in 5 years 3. Birth after 2008 4. Child is alive for at least one year Exclusion criteria (any of the following) 1. Birth after July 2011 (at least 1-year follow-up period)	Outcome component (threshold: 4) 1. Child received BCG at once 2. Child received polio vaccine at least 3 times 3. Child received DPT vaccine at least 3 times 4. Child received measles vaccine at once
Malaria prevention	Inclusion criteria (all of the following) 1. 15–49 years old 2. Have at least one birth in 5 years 3. Child is alive	Outcome component (threshold: 2) 1. Slept under treated mosquito net the previous night 2. Mother slept under the mosquito net 3. Child slept under the mosquito net

The mothers were judged to have access to qualified health services if they could access more domains than the threshold for each health service

*Abbreviations*: *ANC* Antenatal care, *BCG* Bacillus Calmette- Guérin, *DPT* Diphtheria, pertussis, and tetanus

### Exposure

We measured the acute damage of the earthquake and classified it into two dimensions: household damage and regional damage. Household impact was defined as the housing damage (yes or no) and/or loss of a family member (yes or no) due to the earthquake. If mothers experienced housing damage and/or loss of a family member due to the earthquake, they were defined as having suffered household damage. We measured both based on the retrospective self-report by mothers who answered the Haiti sixth DHS questionnaire. Regional damage was defined as living in the region where 50% or more of houses had been damaged. This definition was used in the previous study that explored the impact of the 2010 Haiti earthquake on birth outcomes [8]. To the best of our knowledge, there is no established way to define the damage from earthquake to essential health services in low- and middle-income countries. Therefore, we created several definitions of the earthquake damage combining two dimensions of the earthquake impact (household damage and regional damage). The first, second, and third definitions are based on the similar definition of household and regional damage, but consider different combinations (first: household damage or regional damage; second: household damage; third: regional damage).

First, as a primary definition, mothers who had experienced at least one of household damage or regional damage were defined as the exposed group. Second, mothers who had suffered household damage were defined as exposed regardless of their exposure to regional damage. Third, mothers who had experienced regional damage were defined as exposed regardless of their exposure to household damage. To measure short-term damage from the earthquake in antenatal care, delivery care, and vaccination, we limited the exposed group to mothers who reported a live birth within six months after the earthquake.

### Outcomes

The outcomes were: access to quality health services in family planning, antenatal care, delivery care, child vaccination, and malaria prevention because these are considered essential health services under UHC [10] and could be calculated using the sixth Haiti DHS datasets. We conducted separate analyses for each health service, and the cohort of each analysis was mothers who needed them. Table 1 presents the definitions of the cohort, and the quality health services for each health service. We defined quality health services based on the number of components of essential health services provided to women under the umbrella of each health care area (family planning, antenatal care, delivery care, vaccination, and malaria prevention). The components of essential health services that should be provided to women to assure quality were defined following international guidelines and previous papers: DHS Revising Unmet Need for Family Planning [11], the WHO family planning guideline of 2018 [12] and antenatal care guideline of 2016 [13], previous studies on antenatal care [14, 15], WHO intrapartum care guideline of 2018 [16], WHO immunization guideline of 2020 for vaccination [17], and the DHS report for malaria prevention and treatment [18]. This approach to assessing the coverage of quality health services by counting the accessed components of essential health services in each health service area is known as effective coverage (EC), which measures the efforts under UHC to accurately reflect the access to quality health services, and was developed in response to UHC measuring only access to the health services previously, and not their quality [19]. While crude coverage simply includes the fraction of those who have access to health services, regardless of service quality, EC further considers the quality of these health services by counting only those who have access to such service quality, to measure health service coverage [20]. In our study, the mothers were judged to have access to quality health services if they could access more components for each health service than the threshold of 3 out of 4 domains for family planning, 5 out of 8 for antenatal care, 4 out of 7 for delivery care, 4 out of 4 for vaccination, 2 out of 3 for malaria prevention. For example, if the mothers had accessed more than 5 components in antenatal care, she was considered to have the access to quality health services in antenatal care.

### Covariates

We assessed several individual-level characteristics of mothers: age (continuous variable), smoking status (yes or no), education status (no education, primary, secondary, or higher), urban or rural, region of residence (Aire Metropolitaine/ Reste-Ouest, Artibonite, Camps, Centre, Grand'Anse, Nippes, Nord, Nord-Est, Nord-Ouest, Sud, Sud-Est), decision maker for health care (not mother or mother), household wealth (poor, middle, rich), alcohol drinking (everyday, time to time, rarely, never), mothers’ occupation (not working, non-professional, agricultural, professional), and fathers’ occupation (not working, non-professional, agricultural, professional).

### Statistical analysis

Mothers’ characteristics were described as the mean and SD for continuous variables and as the number and percentage for categorical variables. These characteristics were compared between the earthquake exposed group and the unexposed group stratified by before and after earthquake using Mann–Whitney test for continuous variables and Fisher’s exact test for categorical variables.

For antenatal care, delivery care, and vaccination, we used the difference-in-difference (DID) analysis. The DID design requires two differences: the difference in the outcomes comparing after and before the event in the group exposed to the event (A1), and the same difference in the group unexposed to the event (A2). The change in outcomes associated with the event unexplained by the secular trends could be estimated as A1–A2. In our study, the effect of the Haiti 2010 earthquake on EC in each health service was analyzed using the logistic regression model and the equation below:

$$logit\Pr{\left[{Y^i=Exposure^i,\;Postperiod^i,\;C_i}\right]}=\beta0+\beta1\times Exposure^i+\beta2\times Postperiod^i+\beta3\times Exposure^i\times Postperiod^i+\beta4\times C^i$$

where [1] PR*ί* is the possibility of experiencing the outcome of interest for participant *i*; (2) *Exposure*_*ί*_ is 1 if the participant *i* experienced the earthquake; (3) *Postperiod*_*ί*_ is 1 if the participant *i* gave birth after the earthquake; (4) the interaction between *Exposure*_*ί*_ and *Postperiod*_*ί*_ captures the effect of the earthquake (in the logit scale) on outcomes of interest after excluding the effect of temporal trend on the outcomes; (5) *C*_*ί*_ is the vector notation of mothers’ age and education status.

In the DID analysis, we included mothers’ age and education status and did not include other covariates because the latter were measured after the earthquake and were probably affected by it; hence, if they were adjusted, the effect estimate would have been underestimated. To evaluate the validity of the DID design, we examined pre-earthquake trends in the outcomes and assessed the plausibility of the parallel trend assumption [21]. For family planning and malaria prevention, we conducted multivariate logistic regression analysis because, for these health services, DHS did not record the data before the earthquake. In these analyses, we adjusted for variables as in the DID analysis.

For both DID and multivariable logistic regression analyses, we applied a mixed effect model with the random effect of region (11 regions in Haiti). To address potential bias due to missing data, we applied the multiple imputation method in which we used the chained equations to create 10 imputed datasets (mice, or multivariate imputation by chained equations, in R). The estimates from the 10 imputed datasets were then combined using Rubin’s rules [22].

We conducted several sensitivity analyses. First, for the outcome of antenatal care, we excluded births within 10 months after the earthquake because these mothers likely received antenatal care both before and after the earthquake; hence, the outcomes may be misclassified in terms of the temporal relationship with the exposure. Second, we conducted DID and multiple regression analyses with the outcomes in continuous variable (the number of domains where mothers accessed each health service in Table 1) instead of binary variable, considering that the threshold definitions for each outcome would lead to measurement bias due to the misclassification.

All statistical analyses were two-tailed and conducted using R, Version 4.0.3 and Oracle® R Enterprise, Version 1.4.1 (Oracle, Redwood Shores, CA, USA). All methods were carried out in accordance with relevant guidelines and regulations.

## Results

The final sample included 5703 mothers; 3229 reported a live birth before the earthquake and 1844 reported a live birth after. The earthquake-exposed mothers were more likely to live in urban area, have higher education and household wealth status, and have non-agricultural occupations (Table 2).

**Table 2:** Mothers’ backgrounds

	Before earthquake			After earthquake		
	Unexposed	Exposed	*P*-value	Unexposed	Exposed	*P*-value
n	1580	1649		980	864	
Age (mean (SD))	36.6 (8.0)	36.1 (8.0)	0.065	30.1 (7.0)	29.1 (7.0)	0.004
Smoking status (%)			0.313			0.012
No	1527 (96.6)	1577 (95.6)		968 (98.8)	837 (96.9)	
Yes	52 (3.3)	70 (4.2)		12 (1.2)	25 (2.9)	
Education status (%)			< 0.001			< 0.001
No education	492 (31.1)	406 (24.6)		276 (28.2)	167 (19.3)	
Primary	656 (41.5)	635 (38.5)		442 (45.1)	373 (43.2)	
Secondary	393 (24.9)	559 (33.9)		236 (24.1)	297 (34.4)	
Higher	39 (2.5)	49 (3.0)		26 (2.7)	27 (3.1)	
Urban (%)	556 (35.2)	963 (58.4)	< 0.001	229 (23.4)	407 (47.1)	< 0.001
Region of residence (%)			< 0.001			< 0.001
Aire Metropolitaine/Reste-Ouest	11 (0.7)	796 (48.3)		4 (0.4)	377 (43.6)	
Artibonite	200 (12.7)	71 (4.3)		103 (10.5)	44 (5.1)	
Camps	2 (0.1)	352 (21.3)		0 (0.0)	212 (24.5)	
Centre	183 (11.6)	53 (3.2)		137 (14.0)	30 (3.5)	
Grand'Anse	126 (8.0)	67 (4.1)		88 (9.0)	46 (5.3)	
Nippes	157 (9.9)	84 (5.1)		61 (6.2)	25 (2.9)	
Nord	235 (14.9)	27 (1.6)		142 (14.5)	13 (1.5)	
Nord-Est	219 (13.9)	10 (0.6)		152 (15.5)	6 (0.7)	
Nord-Ouest	189 (12.0)	44 (2.7)		117 (11.9)	24 (2.8)	
Sud	159 (10.1)	48 (2.9)		109 (11.1)	35 (4.1)	
Sud-Est	99 (6.3)	97 (5.9)		67 (6.8)	52 (6.0)	
Health care decision (%)			0.094			0.139
By others	341 (21.6)	321 (19.5)		274 (28.0)	210 (24.3)	
By mother	947 (59.9)	979 (59.4)		614 (62.7)	558 (64.6)	
Household wealth (%)			< 0.001			< 0.001
Poor	727 (46.0)	425 (25.8)		615 (62.8)	288 (33.3)	
Middle	323 (20.4)	483 (29.3)		168 (17.1)	279 (32.3)	
Rich	530 (33.5)	741 (44.9)		197 (20.1)	297 (34.4)	
Drinking alcohol (%)			0.043			0.338
Every day	8 (0.5)	10 (0.6)		2 (0.2)	3 (0.3)	
Time to time	58 (3.7)	50 (3.0)		13 (1.3)	18 (2.1)	
Rarely	250 (15.8)	328 (19.9)		130 (13.3)	132 (15.3)	
Never	1263 (79.9)	1260 (76.4)		834 (85.1)	711 (82.3)	
Mother's occupation (%)			< 0.001			< 0.001
Not working	477 (30.2)	558 (33.8)		409 (41.7)	413 (47.8)	
Non-professional	862 (54.6)	937 (56.8)		450 (45.9)	392 (45.4)	
Agricultural	181 (11.5)	104 (6.3)		103 (10.5)	40 (4.6)	
Professional	60 (3.8)	50 (3.0)		18 (1.8)	18 (2.1)	
Father's occupation (%)			< 0.001			< 0.001
Not working	14 (0.9)	26 (1.6)		7 (0.7)	9 (1.0)	
Non-professional	529 (33.5)	833 (50.5)		284 (29.0)	466 (53.9)	
Agricultural	805 (50.9)	482 (29.2)		568 (58.0)	241 (27.9)	
Professional	188 (11.9)	265 (16.1)		85 (8.7)	122 (14.1)	

**Fig. 1:**
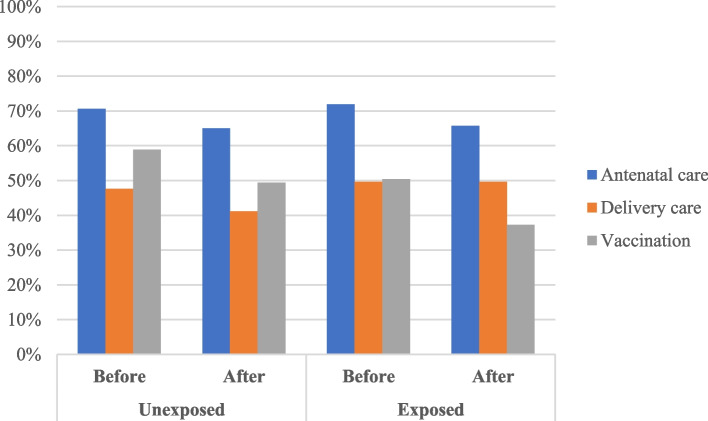
Percentage of mothers who had access to qualified health services. Legend: Exposed group were those who suffered at least regional or household damage of the earthquake. If the mothers had births after the earthquake, they were categorized as after earthquake group. The bar graph represents the percentages of mothers who could access qualified health services. Family planning and malaria prevention were not listed in the figure because, for those health services, access before the earthquake was not measured

**Fig. 2:**
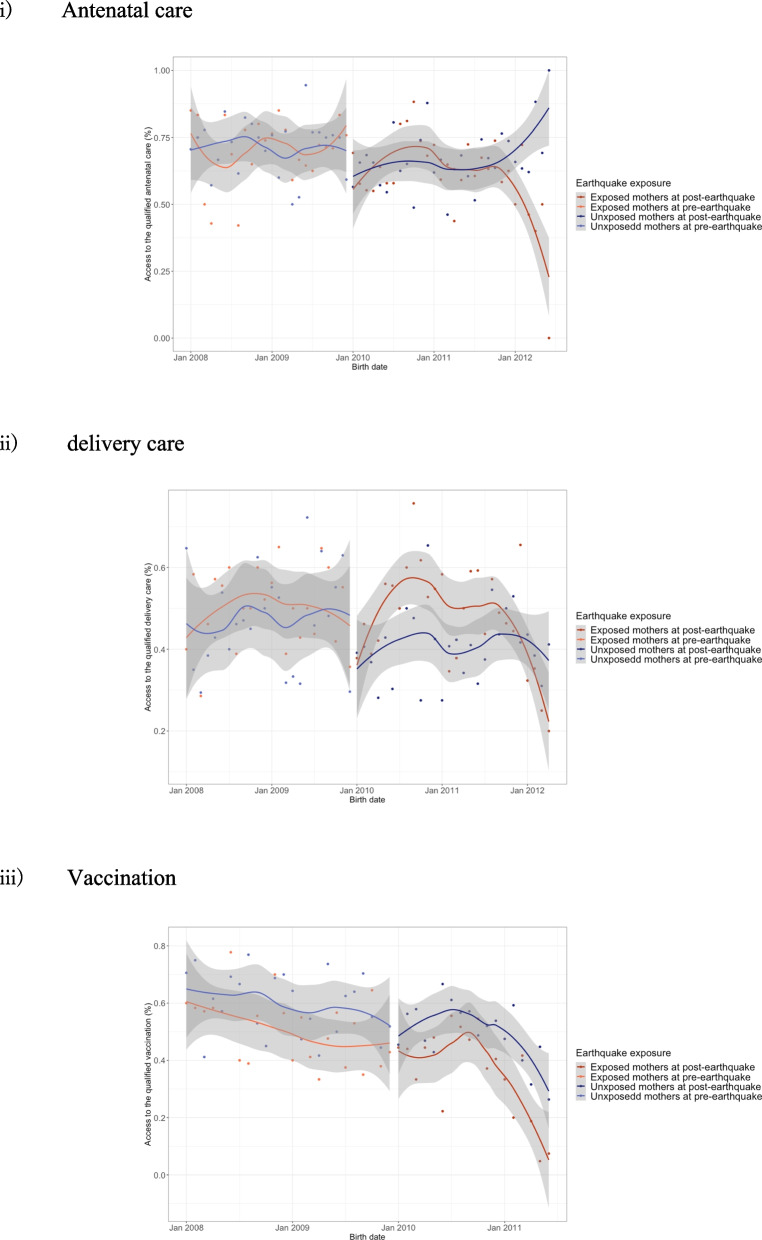
Trend of access to qualified health services before and after the earthquake in

The difference between pre- and post-earthquake access to quality health services was -5.6% for mothers without acute earthquake damage (mothers who did not suffer from household damage or not live in hugely damaged areas) and -6.2% for mothers with acute earthquake damage (mothers who suffered from household damage or lived in hugely damaged areas) in antenatal care, -6.5% and 0% in delivery care, and -9.5% and -13.1% in vaccination, respectively (Table 3 and Fig. 1). Also, in almost all components of each essential health service, access to quality health services decreased from pre-earthquake (Table 4). Figure 2 shows the outcome trend in mothers with/without acute earthquake damage were parallel before the earthquake for antenatal care, delivery care, and vaccination. Table 3 shows the results of the DID and multivariable logistic regression analyses. In unadjusted and adjusted DID analyses, there was no strong evidence of the associations between the exposure to acute earthquake damage and access to quality antenatal care, delivery care, and vaccination. Similar results were confirmed in unadjusted and adjusted multivariable logistic regression analyses in family planning and malaria prevention. Table 5 shows the results of the additional and sensitivity analyses. We observed similar tendency in all sensitivity analyses.

**Table 3:** Results of difference-in-difference analyses and multivariable logistic regression analyses

	Before-After Difference		Unadjusted analyses		Adjusted analyses	
	Unexposed % (n)	Exposed % (n)	Odds ratio (95% CI)	*P*-value	Odds ratio (95% CI)	*P*-value
Family planning	33.5% (413)	25.4% (283)	0.95 (0.74–1.24)	0.717	0.96 (0.73–1.26)	0.777
Antenatal care	70.6%-65.0% (338–562)(-5.6%)	71.9%-65.7% (348–644)(-6.2%)	1.05 (0.74–1.48)	0.785	0.97 (0.68–1.38)	0.857
Delivery care	47.6%-41.1% (226–388)(-6.5%)	49.6%-49.6% (233–412)(0%)	1.22 (0.87–1.71)	0.244	1.30 (0.94–1.79)	0.108
Vaccination	58.9%-49.4% [279–288](-9.5%)	50.4%-37.3% (235–191)(-13.1%)	0.87 (0.61–1.24)	0.431	0.82 (0.57–1.18)	0.282
Malaria prevention	17.4% (266)	21.8% (306)	0.86 (0.64–1.16)	0.322	0.88 (0.65–1.18)	0.384

In the adjusted analyses, the covariates included were mothers’ age and education status

**Table 4:** Before-after difference of qualified health service coverage in component level

	Unexposed			Exposed		
	Before	After	Difference	Before	After	Difference
**Family planning (n)**	1233			1116		
Using modern contraceptive method = Yes (%)	-	598 (48.5)	-	-	438 (39.2)	-
Told about side effect = Yes (%)	-	441 (35.8)	-	-	299 (26.8)	-
Told how to deal with side effects = Yes (%)	-	386 (31.3)	-	-	270 (24.2)	-
Told about other method = Yes (%)	-	369 (29.9)	-	-	238 (21.4)	-
**Antenatal care (n)**	479	864		484	980	
First ANC visit in up to 12 weeks = Yes (%)	158 (33.1)	234 (27.1)	-6.0%	151 (31.2)	240 (24.5)	-6.7%
More than 4 times ANC visits = Yes (%)	336 (70.6)	560 (65.0)	-5.6%	349 (72.4)	635 (64.9)	-7.5%
Talked about the signs of complication = Yes (%)	295 (62.2)	512 (59.5)	-2.7%	287 (59.5)	585 (59.8)	0.3%
Blood pressure taken during pregnancy = Yes (%)	411 (86.3)	750 (87.1)	0.8%	440 (91.3)	838 (85.6)	-5.7%
Urine sample taken during pregnancy = Yes (%)	352 (73.9)	629 (73.1)	-0.8%	378 (78.4)	711 (72.6)	-5.8%
Blood sample taken during pregnancy = Yes (%)	358 (75.2)	644 (74.8)	-0.4%	381 (79.0)	710 (72.5)	-6.5%
Drugs for intestinal worms during pregnancy = Yes (%)	82 (17.2)	133 (15.4)	-1.8%	92 (19.0)	157 (16.0)	-3.0%
Iron tablet during pregnancy = Yes (%)	353 (74.0)	624 (72.2)	-1.8%	386 (79.8)	735 (75.0)	-4.8%
**Delivery care (n)**	475	945		470	831	
Mother checked after delivery (%)	167 (35.2)	284 (30.1)	-5.1%	208 (44.3)	355 (42.8)	-1.5%
Baby checked within 1 h (%)	29 (6.1)	22 (2.3)	-3.8%	33 (7.1)	51 (6.1)	-1.0%
At least 24 h facility stay (%)	129 (27.2)	196 (20.7)	-6.5%	135 (28.9)	263 (31.8)	2.9%
Baby checked within 2 months (%)	326 (68.6)	544 (57.6)	-11.0%	345 (73.7)	532 (64.1)	-9.6%
Received Vitamin A (%)	258 (54.3)	414 (43.9)	-10.4%	245 (52.2)	384 (46.3)	-5.9%
Ever breastfeed (%)	464 (97.7)	932 (98.6)	0.9%	453 (96.4)	803 (96.9)	0.5%
Put baby to mother's breast within 60 min (%)	285 (61.7)	660 (71.0)	9.3%	229 (50.7)	501 (62.6)	11.9%
**Vaccination (n)**	474	583		466	512	
BCG vaccine = Yes (%)	419 (88.4)	488 (84.1)	-4.30%	395 (86.1)	409 (81.3)	-4.80%
DPT vaccine 3 or more = Yes (%)	363 (76.6)	372 (64.1)	-12.50%	313 (68.8)	292 (57.9)	-10.90%
Polio vaccine 3 or more = Yes (%)	333 (70.3)	362 (62.4)	-7.90%	291 (63.4)	276 (54.7)	-8.70%
Measles vaccine = Yes (%)	369 (78.5)	402 (69.6)	-8.90%	328 (71.8)	266 (52.7)	-19.10%
**Malaria prevention (n)**	1528			1406		
Family slept under the treated net last night = Yes (%)	-	252 (16.5)	-	-	276 (19.6)	-
Mother slept under the mosquito net last night = Yes (%)	-	274 (17.9)	-	-	311 (22.1)	-
Child slept under the mosquito net last night = Yes (%)	-	204 (13.4)	-	-	249 (18.0)	-

*Abbreviations*: *ANC* Antenatal care, *BCG* Bacillus Calmette- Guérin, *DPT* Diphtheria, pertussis, and tetanus

**Table 5:** Results of additional analyses

	Household damage		Regional damage		Short-term damage		Without birth within 10 months after the earthquake		Outcome as continuous variable	
	Odds ratio (95% CI)	*P*-value	Odds ratio (95% CI)	*P*-value	Odds ratio (95% CI)	*P*-value	Odds ratio (95% CI)	*P*-value	Coefficient (95% CI)	*P*-value
Family planning	0.93 (0.75–1.17)	0.549	0.62 (0.38–1.00)	0.052	-		-		-0.05 (-0.24–0.13)	0.590
Antenatal care	1.03 (0.71–1.48)	0.893	1.06 (0.72–1.55)	0.762	0.80 (0.48–1.35)	0.408	0.85 (0.58–1.25)	0.413	0.14 (-0.19–0.46)	0.408
Delivery care	1.09 (0.77–1.54)	0.616	1.35 (0.95–1.93)	0.097	1.15 (0.69–1.90)	0.601	-		0.15 (-0.06–0.37)	0.161
Vaccination	0.88 (0.60–1.28)	0.500	0.93 (0.63–1.36)	0.697	0.82 (0.50–1.34)	0.428	-		-0.15 (-0.38–0.09)	0.226
Malaria prevention	1.19 (0.95–1.49)	0.125	1.25 (0.73–2.15)	0.415	-		-		-0.04 (-0.15–0.06)	0.44

Household damage: mothers were defined as exposed if she experienced housing damage or loss of family member; Regional damage: mothers were defined as exposed if 50% of households in the region where mothers lived were exposed to the earthquake; Short-term damage: limited the earthquake exposed group to mothers who had the birth within 6 months after the earthquake; Without birth within 10 months after the earthquake: excluded mothers who had birth within 10 months since the earthquake; Outcome as continuous variable: d outcomes as continuous variables

## Discussion

We conducted this retrospective study to explore the impact of the 2010 Haiti earthquake on quality health services in mothers. Contrary to our hypothesis, we did not observe strong evidence that mothers with acute damage from the earthquake experienced lower access to quality health services compared to mothers without it. This may be because the acute earthquake damage did not cause any negative impact, or the earthquake equally impacted both groups. Because both groups of mothers' access to quality health services were found to have deteriorated in the descriptive analyses (Table 3), the earthquake might have worsened the access to quality health services for mothers with/without acute earthquake damage.

To the best of our knowledge, this is the first study that shows that mothers who did not suffer from household damage or did not live in hugely damaged areas also experienced deterioration of access to quality health services. Previous studies show that mothers exposed to the earthquake were more likely to have adverse reproductive health outcomes compared to unexposed or less exposed mothers [6–8]. This inconsistency may come from the differences in outcome and exposure definitions between these studies. In our study, the outcome of interest was quality of health services while previous studies focused on access regardless of quality. Additionally, for a more comprehensive measurement of earthquake exposure, we combined household and regional damage of the earthquake, while previous studies focused narrowly on living in an area with greater housing damage or displacement.

There could be several explanations for the deterioration of access to quality health services even for mothers who did not suffer from household damage or did not live in hugely damaged areas. First, long-term structural damage on the health care system may have adversely affected the access to quality health services even for mothers who avoided acute earthquake damage. Well-known long-term structural damages involve the spread of communicable diseases. Even before the earthquake, it was only less than 10% of the population who had access to potable tap water, and less than one-third that had access to electricity [23]. However, the earthquake caused a decrease in air or water quality and a lack of food safety or sanitation after the earthquake [24]. After the 2010 earthquake, Haiti experienced one of the largest cholera outbreaks, causing 600,000 cases and more than 7,000 deaths in the first two years after the earthquake [25]. This outbreak was widespread, including rural areas that were far from the epicenter of the earthquake [26]. In addition to the deterioration of clean water and food supply, the earthquake brought the collapse of multiple infrastructures in Haiti. It destroyed more than 180 government buildings and 13 among 15 key government offices [23]. Haiti’s Ministry of Health lost more than 200 staff in the earthquake and a large part of its operating capacity was devastated [27]. Even before the earthquake, health facilities in Haiti suffered from a high turnover rate and inadequately trained staff [27]. It is reasonable to think that the earthquake further devastated the supply of well-trained staff in urban and rural health facilities. Therefore, even though mothers in rural areas could avoid acute earthquake damage, it was highly likely that they suffered due to the long-term structural damages resulting in deteriorated access to essential health services.

Second, mothers who did not suffer from household damage or did not live in hugely damaged areas were relatively more exposed to the long-term structural damages due to pre-existing vulnerabilities. In our study, mothers who did not suffer from household damage or did not live in hugely damaged areas mainly lived in rural areas and had lower education or wealth status than the exposed mothers. There is evidence that poorer and less educated people are more vulnerable to the disaster [28, 29]. Thus in Haiti, the earthquake disproportionately affected poor people in rural areas and worsened their access to quality health services, irrespective of their exposure to acute earthquake damages.

Third, although mothers who suffered from household damage or lived in hugely damaged areas suffered the most (including long-term structural damage), the overall effect could have been mitigated slightly due to the intensive foreign aid and reconstruction efforts in urban areas, compared to mothers without acute earthquake damage as they were mainly in rural areas [6].

Our study has several strengths. First, we used the large-scale nationally representative household data, which were constructed with well-established sampling and data collection methods. Second, we applied DID, a statistically robust natural experimental method, which could appropriately consider unmeasured non-time varying confounders. Third, multiple analyses with different definitions of earthquake exposure showed similar tendencies, strengthening the robustness of our results.

Alongside this, several limitations should be acknowledged. First, our study targeted mothers in Haiti, and the generalizability of our results to mothers in other countries should be carefully interpreted. Second, if mothers suffered other impacts at the same time as the earthquake, the DID method could not appropriately estimate the impact of the earthquake (common shock assumption) [30]. However, an earthquake is a sudden and unexpected event; therefore, we believe there was no common shock that impacted mothers in Haiti at the same time as the earthquake. Third, our definition of the regional impact of the earthquake may have not fully reflected the full impact as it considered only the percentage of housing damage in each area. Damage to health care workers, medical equipment or drug supply, and funds for health care should also be considered for a more accurate measurement of the regional damage. Fourth, DHS is a cross-sectional survey that retrospectively collected information on women’s characteristics, earthquake exposure, and health service use. If women who were exposed to the earthquake tended to remember their worsened access to health services, the results of this study were affected by measurement bias away from the null. Also, a cross-sectional survey made it difficult to collect information on women’s characteristics before the earthquake. Matching between exposed and unexposed by these baseline characteristics would reduce the bias by unmeasured confounders.

## Conclusions

The 2010 earthquake negatively affected Haitian women’s access to quality essential health services regardless of their exposure to acute earthquake damage. Furthermore, mothers who did not suffer from household damage or did not live in hugely damaged areas may have suffered from the long-term structural damages of the earthquake, which were likely exacerbated by other structural factors such as their lower education and economic status. As part of recovery efforts for UHC in Haiti, government and emergency aid need to pay attention not only to mothers who suffered from acute earthquake damage, but also to mothers who did not but were nonetheless exposed to long-term structural damages and rendered vulnerable. Further studies that use longitudinal data with robust balancing methods such as matching are required.

## Data Availability

The data utilized for the present study is freely available in the public domain through: https://www.dhsprogram.com/methodology/survey/survey-display-368.cfm

## Abbreviations

UHC: Universal Health Coverage
WHO: World Health Organization
DHS: Demographic and Health Survey
EC: Effective coverage
DID: Difference-in-difference
